# Complete genome sequence of *Sulfurimonas autotrophica* type strain (OK10^T^)

**DOI:** 10.4056/sigs.1173118

**Published:** 2010-10-27

**Authors:** Johannes Sikorski, Christine Munk, Alla Lapidus, Olivier Duplex Ngatchou Djao, Susan Lucas, Tijana Glavina Del Rio, Matt Nolan, Hope Tice, Cliff Han, Jan-Fang Cheng, Roxanne Tapia, Lynne Goodwin, Sam Pitluck, Konstantinos Liolios, Natalia Ivanova, Konstantinos Mavromatis, Natalia Mikhailova, Amrita Pati, David Sims, Linda Meincke, Thomas Brettin, John C. Detter, Amy Chen, Krishna Palaniappan, Miriam Land, Loren Hauser, Yun-Juan Chang, Cynthia D. Jeffries, Manfred Rohde, Elke Lang, Stefan Spring, Markus Göker, Tanja Woyke, James Bristow, Jonathan A. Eisen, Victor Markowitz, Philip Hugenholtz, Nikos C. Kyrpides, Hans-Peter Klenk

**Affiliations:** 1 DSMZ - German Collection of Microorganisms and Cell Cultures GmbH, Braunschweig, Germany; 2 DOE Joint Genome Institute, Walnut Creek, California, USA; 3 Los Alamos National Laboratory, Bioscience Division, Los Alamos, New Mexico, USA; 4 HZI – Helmholtz Centre for Infection Research, Braunschweig, Germany; 5 Biological Data Management and Technology Center, Lawrence Berkeley National Laboratory, Berkeley, California, USA; 6 Oak Ridge National Laboratory, Oak Ridge, Tennessee, USA; 7 University of California Davis Genome Center, Davis, California, USA

**Keywords:** mesophilic, facultatively anaerobic, sulfur metabolism, deep-sea hydrothermal vents, spermidine, Gram-negative, *Helicobacteriaceae*, *Epsilonproteobacteria*, GEBA

## Abstract

*Sulfurimonas autotrophica* Inagaki *et al*. 2003 is the type species of the genus *Sulfurimonas*. This genus is of interest because of its significant contribution to the global sulfur cycle as it oxidizes sulfur compounds to sulfate and by its apparent habitation of deep-sea hydrothermal and marine sulfidic environments as potential ecological niche. Here we describe the features of this organism, together with the complete genome sequence and annotation. This is the second complete genome sequence of the genus *Sulfurimonas* and the 15^th^ genome in the family *Helicobacteraceae*. The 2,153,198 bp long genome with its 2,165 protein-coding and 55 RNA genes is part of the *** G****enomic* *** E****ncyclopedia of* *** B****acteria and* *** A****rchaea * project.

## Introduction

Strain OK10^T^ (= DSM 16294 = ATCC BAA-671 = JCM 11897) is the type strain of *Sulfurimonas autotrophica* [1], which is the type species of its genus *Sulfurimonas* [1,2]. Together with *S. paralvinellae* and *S. denitrificans*, the latter of which was formerly classified as *Thiomicrospira denitrificans* [3]. There are currently three validly named species in the genus *Sulfurimonas* [4,5]. The autotrophic and mixotrophic sulfur-oxidizing bacteria such as the members of the genus *Sulfurimonas* are believed to contribute significantly to the global sulfur cycle [6]. The genus name derives from the Latin word ‘*sulphur*’, and the Greek word ‘*monas*’, meaning a unit, in order to indicate a “sulfur-oxidizing rod” [1]. The species epithet derives from the Greek word ‘auto’, meaning self, and from the Greek adjective ‘*trophicos*’ meaning nursing, tending or feeding, in order to indicate its autotrophy [1]. *S. autotrophica* strain OK10^T^, like *S. paralvinellae* strain GO25^T^ (= DSM 17229), was isolated from the surface of a deep-sea hydrothermal sediment on the Hatoma Knoll in the Mid-Okinawa Trough hydrothermal field [1,2]. Thus, the members of the genus *Sulfurimonas* appear to be free living, whereas the other members of the family *Helicobacteraceae*, the genera *Helicobacter* and *Wolinella*, appear to be strictly associated with the human stomach and the bovine rumen, respectively. Here we present a summary classification and a set of features for *S. autotrophica* OK10^T^, together with the description of the complete genomic sequencing and annotation.

## Classification and features

There exist currently no experimental reports that indicate further cultivated strains of this species. The type strains of *S. denitrificans* and *S. paralvinellae* share 93.5% and 96.3% 16S rRNA gene sequence similarity with strain OK10^T^. Further analysis also revealed that strain OK10^T^ shares high similarity (99.1%) with the uncultured clone sequence PVB-12 (U15104) obtained from a microbial mat near the deep-sea hydrothermal vent in the Loihi Seamont, Hawaii [7]. This further corroborates the distribution of *S. autotrophica* in hydrothermal vents. The 16S rRNA gene sequence similarities of strain OK10^T^ to metagenomic libraries (env_nt) were 87% or less, indicating the absence of further members of the species in the environments screened so far (status August 2010).

**Figure 1:**
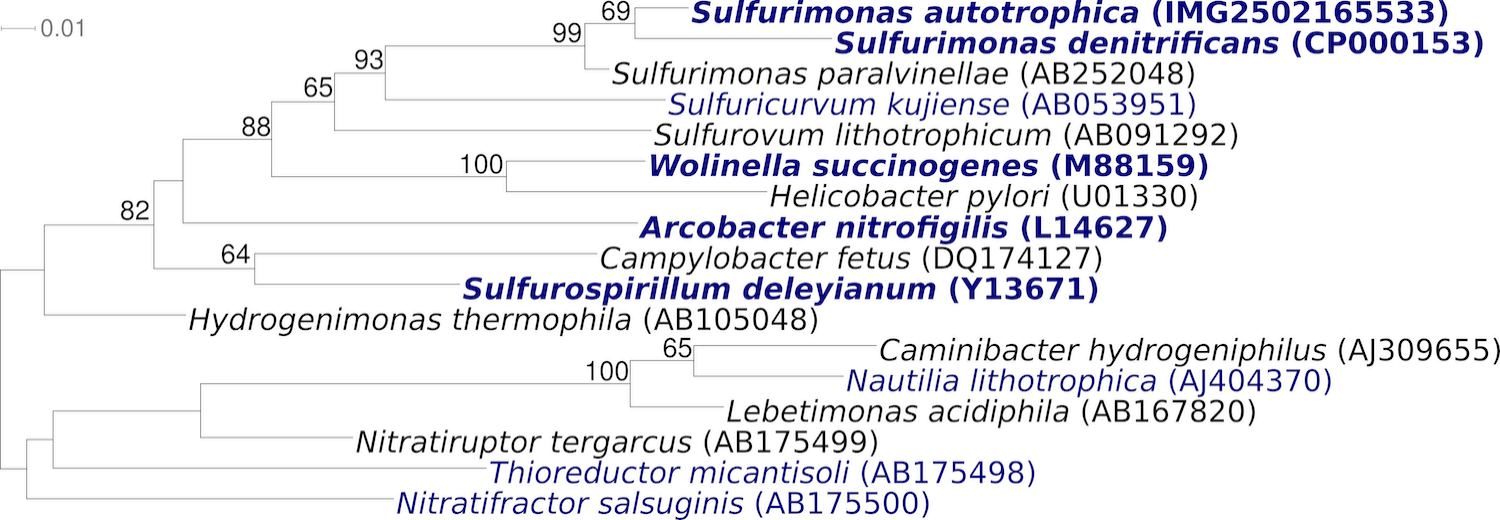
Phylogenetic tree highlighting the position of *S. autotrophica* OK10^T^ relative to the type strains of the other species within the genus and the type strains of the other genera within the order *Campylobacterales*. The tree was inferred from 1,327 aligned characters [8,9] of the 16S rRNA gene sequence under the maximum likelihood criterion [10] and rooted in accordance with current taxonomy [11]. The branches are scaled in terms of the expected number of substitutions per site. Numbers above branches are support values from 350 bootstrap replicates [12] if larger than 60%. Lineages with type strain genome sequencing projects registered in GOLD [13] are shown in blue, published genomes in bold [14,15], such as the recently published GEBA genomes from *Sulfurospirillum deleyianum* [16] and *Arcobacter nitrofigilis* [17].

Figure 1 shows the phylogenetic neighborhood of *S. autotrophica* OK10^T^ in a 16S rRNA based tree. The sequences of the four 16S rRNA gene copies in the genome differ from each other by up to four nucleotides, and differ by up to three nucleotides from the previously published sequence (AB088431).

**Figure 2:**
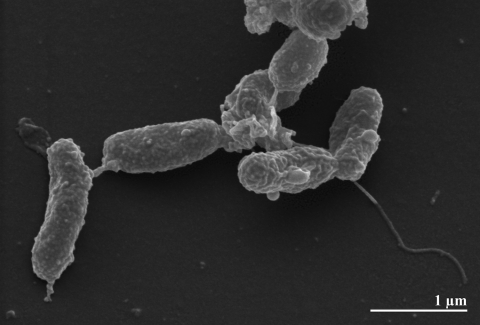
Scanning electron micrograph of *S. autotrophica* OK10^T^

The cells of strain OK10^T^ are Gram-negative, occasionally slightly curved rods of 1.5–2.5 × 0.5-1.0 µm (Figure 2 and Table 1) [1]. On solid medium, the cells form white colonies [1]. Under optimal conditions, the generation time of *S. autotrophica* strain OK10^T^ is approximately 1.4 h [1,2]. The reductive tricarboxylic acid (rTCA) cycle for autotrophic CO_2_ fixation is present in strain OK10^T^, as shown by PCR amplification of the respective genes [28]. Moreover, the activities of several rTCA key enzymes (ACL, ATP dependent citrate lyase; POR, pyruvate:acceptor oxidoreductase; OGOR, 2-oxoglutarase:accecptor oxidoreductase; ICDH, isocytrate dehydrogenase) have been determined, also in comparison to *S. paralvinellae* and *S. denitrificans* [28]. There were no enzyme activities for the phosphoenolpyruvate and ribulose 1,5-bisphosphate (Calvin-Benson) pathways detected in strain OK10^T^ [28], though the latter is apparently active in *S. thermophila* [28]. Also, soluble hydrogenase activity was not found in strain OK10^T^ [28]. With respect to sulfur oxidation, enzyme activity for SOR (sulfite oxidoreductase) but not for APSR (adenosine 5′-phosphate sulfate reductase) and TSO (thiosulfate-oxidizing enzymes) were detected [28]. A detailed comparison of these enzyme activities to *S. paralvinellae* and *S. denitrificans* is given in Takai *et al.* [28]. Elemental sulfur, thiosulfate or sulfide is utilized as the sole electron donor for chemolithoautotrophic growth with O_2_ as electron acceptor. Thereby thiosulfate is oxidized to sulfate [1]. Organic substrates and H_2_ are not utilized as electron donors and only oxygen is utilized as an electron acceptor [28]. Strain OK10^T^ requires 4% sea salt for growth [1] and is not able to reduce nitrate [2].

**Table 1:** Classification and general features of *S. autotrophica* OK10^T^ according to the MIGS recommendations [18]

**MIGS ID**	**Property**	**Term**	**Evidence code**
	Current classification	Domain *Bacteria*	TAS [19]
		Phylum *Proteobacteria*	TAS [20]
		Class *Epsilonproteobacteria*	TAS [21,22]
		Order *Campylobacterales*	TAS [23,24]
		Family *Helicobacteraceae*	TAS [24,25]
		Genus *Sulfurimonas*	TAS [1,2]
		Species *Sulfurimonas autotrophica*	TAS [1]
		Type strain OK10	TAS [1]
	Gram stain	negative	TAS [1]
	Cell shape	short rods, occasionally slightly curved rods	TAS [1]
	Motility	by monotrichous, polar flagellum	TAS [1]
	Sporulation	non-sporulating	TAS [1]
	Temperature range	10°C - 40°C	TAS [1]
	Optimum temperature	23°C - 26°C	TAS [1]
	Salinity	4% NaCl	TAS [1]
MIGS-22	Oxygen requirement	aerobic	TAS [1]
	Carbon source	CO_2_	TAS [1]
	Energy source	chemolithoautotrophic, S^0^, Na_2_S_2_O_3_ and Na_2_S x 9H_2_O	TAS [1]
MIGS-6	Habitat	hydrothermal deep-sea sediments	TAS [1]
MIGS-15	Biotic relationship	free living	NAS
MIGS-14	Pathogenicity	not reported	NAS
	Biosafety level	1	TAS [26]
	Isolation	Mid-Okinawa Trough hydrothermal sediments	TAS [1,7]
MIGS-4	Geographic location	Japan, Hatoma Knoll	TAS [1,7]
MIGS-5	Sample collection time	2003 or before	TAS [1]
MIGS-4.1 MIGS-4.2	Latitude Longitude	27.27 127.17	TAS [1]
MIGS-4.3	Depth	sediment surface	TAS [1]
MIGS-4.4	Altitude	not reported	NAS

Evidence codes - IDA: Inferred from Direct Assay (first time in publication); TAS: Traceable Author Statement (i.e., a direct report exists in the literature); NAS: Non-traceable Author Statement (i.e., not directly observed for the living, isolated sample, but based on a generally accepted property for the species, or anecdotal evidence). These evidence codes are from of the Gene Ontology project [27]. If the evidence code is IDA, then it was directly observed by one of the authors or an expert mentioned in the acknowledgements.

### Chemotaxonomy

The major cellular fatty acids found in strain OK10^T^ are C_14:0_ (8.4%), C_16:1_*_cis_* (45.2%), C_16:0_ (37.1%) and C_18:1_*_trans_* (9.4%) [1]. Further fatty acids were not reported [1]. The only polyamine identified in *S. autotrophica* is spermidine [29]. Spermidine was also found in another representative of the order *Campylobacterales*, *Sulfuricurvum kujiense*. For comparison, *Hydrogenimonas thermophila*, the type species and genus of the family *Hydrogenimonaceae* in the order *Campylobacterales*, contains both spermidine and spermine as the major polyamines [29]. The cellular fatty acid composition of *S. autotrophica* was compared with that of other autotrophic *Epsilonproteobacteria* from deep-sea hydrothermal vents: *Nautilia profundicola* AmH^T^, *Lebetimonas acidiphila* Pd55^T^, *Hydrogenimonas thermophila* EP1-55-1%^T^, and *Nitratiruptor tergarcus* MI55-1^T^ [30]. It was found that *S. autotrophica* strain OK10^T^ has much higher levels of the fatty acid C_16:1cis_ (45.2%) than do other *Epsilonproteobacteria* from hydrothermal vents express (3.6%-28.8%) [2,30]. On another hand, the percentage of C_18:1_*_trans_* was the lowest in *S. autotrophica*: (9.4%), while other *Epsilonproteobacteria* contained 20.0%-73.3% [30]. C_14:0_ (8.4%) was also more abundant in strain OK10^T^ than in other strains [30].

## Genome sequencing and annotation

### Genome project history

This organism was selected for sequencing on the basis of its phylogenetic position [31], and is part of the *** G****enomic* *** E****ncyclopedia of* *** B****acteria and* *** A****rchaea * project [32]. The genome project is deposited in the Genome OnLine Database [13] and the complete genome sequence is deposited in GenBank. Sequencing, finishing and annotation were performed by the DOE Joint Genome Institute (JGI). A summary of the project information is shown in Table 2.

**Table 2:** Genome sequencing project information

**MIGS ID**	**Property**	**Term**
MIGS-31	Finishing quality	Finished
MIGS-28	Libraries used	Four genomic libraries: Sanger 8 kb pMCL200 library, 454 pyrosequence standard library, 454 pyrosequence paired end (PE) library, Illumina standard library
MIGS-29	Sequencing platforms	ABI3730, 454 GS FLX Titanium, Illumina GAii
MIGS-31.2	Sequencing coverage	3.7 × Sanger; 121.7 × pyrosequence, 30.0 × Illumina
MIGS-30	Assemblers	Newbler version 2.0.00.20-PostRelease-11-05-2008-gcc-3.4.6, phrap
MIGS-32	Gene calling method	Prodigal 1.4, GenePRIMP
	INSDC ID	CP002205
	Genbank Date of Release	September 15, 2010
	GOLD ID	Gc01373
	NCBI project ID	31347
	Database: IMG-GEBA	2502082114
MIGS-13	Source material identifier	DSM 16294
	Project relevance	Tree of Life, GEBA

### Growth conditions and DNA isolation

*S. autotrophica* strain OK10^T^, DSM 16294, was grown in DSMZ medium 1011 (MJ medium) [33] at 24°C. DNA was isolated from 0.5-1 g of cell paste using MasterPure Gram Positive DNA Purification Kit (Epicenter MGP04100) following the standard protocol as recommended by the manufacturer, with modification st/LALM for cell lysis as described in Wu *et al*. [32].

### Genome sequencing and assembly

The genome was sequenced using a combination of Sanger, 454 and Illumina sequencing platforms. All general aspects of library construction and sequencing can be found at the JGI website (http://www.jgi.doe.gov/). Illumina sequencing data was assembled with VELVET [34], and the consensus sequences were shredded into 1.5 kb overlapped fake reads and used for the assembly with 454 and Sanger data. Contigs resulting from a 454 Newbler (2.0.00.20-PostRelease-11-05-2008-gcc-3.4.6) assembly were shredded into 2 kb fake reads, which were assembled with Sanger data. The Phred/Phrap/Consed software package (www.phrap.com) was used for sequence assembly and quality assessment. After the shotgun stage, reads were assembled with parallel phrap (High Performance Software, LLC). Possible mis-assemblies were corrected with Dupfinisher or transposon bombing of bridging clones (Epicentre Biotechnologies, Madison, WI). Gaps between contigs were closed by editing in Consed, custom primer walk or PCR amplification (Roche Applied Science, Indianapolis, IN) [35]. A total of 790 additional custom primer reactions were necessary to close gaps and to raise the quality of the finished sequence. Illumina reads were also used to improve the final consensus quality using an in-house developed tool - the Polisher [36]. Together, the combination of the Illumina and 454 sequencing platforms provided 155.4 × coverage of the genome. The error rate of the completed genome sequence is less than 1 in 100,000.

### Genome annotation

Genes were identified using Prodigal [37] as part of the Oak Ridge National Laboratory genome annotation pipeline, followed by a round of manual curation using the JGI GenePRIMP pipeline [38]. The predicted CDSs were translated and used to search the National Center for Biotechnology Information (NCBI) nonredundant database, UniProt, TIGRFam, Pfam, PRIAM, KEGG, COG, and InterPro databases. Additional gene prediction analysis and functional annotation was performed within the Integrated Microbial Genomes - Expert Review (IMG-ER) platform [39].

## Genome properties

**Figure 3:**
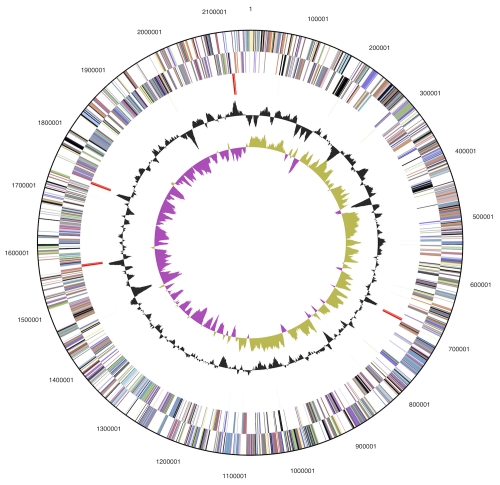
Graphical circular map of the genome. From outside to the center: Genes on forward strand (color by COG categories), Genes on reverse strand (color by COG categories), RNA genes (tRNAs green, rRNAs red, other RNAs black), GC content, GC skew.

The genome consists of a 2,153,198 bp long chromosome with a 35.2% GC content (Table 3 and Figure 3). Of the 2,220 genes predicted, 2,165 were protein-coding genes, and 55 RNAs; seven pseudogenes were also identified. The majority of the protein-coding genes (69.1%) were assigned with a putative function while the remaining ones were annotated as hypothetical proteins. The distribution of genes into COGs functional categories is presented in Table 4.

**Table 3:** Genome Statistics

**Attribute**	**Value**	**% of Total**
Genome size (bp)	2,153,198	100.00%
DNA coding region (bp)	2,043,048	94.88%
DNA G+C content (bp)	758,696	35.24%
Number of replicons	1	
Extrachromosomal elements	0	
Total genes	2,220	100.00%
RNA genes	55	2.48%
rRNA operons	4	
Protein-coding genes	2,165	97.52%
Pseudo genes	7	032%
Genes with function prediction	1,534	69.10%
Genes in paralog clusters	141	6.35%
Genes assigned to COGs	1,590	71.62%
Genes assigned Pfam domains	1,656	74.59%
Genes with signal peptides	429	19.32%
Genes with transmembrane helices	563	25.36%
CRISPR repeats	0	

**Table 4:** Number of genes associated with the general COG functional categories

**Code**	**value**	**%age**	**Description**
J	143	8.1	Translation, ribosomal structure and biogenesis
A	0	0.0	RNA processing and modification
K	70	4.0	Transcription
L	82	4.6	Replication, recombination and repair
B	0	0.0	Chromatin structure and dynamics
D	22	1.2	Cell cycle control, cell division, chromosome partitioning
Y	0	0.0	Nuclear structure
V	30	1.7	Defense mechanisms
T	158	8.9	Signal transduction mechanisms
M	126	7.1	Cell wall/membrane/envelope biogenesis
N	77	4.3	Cell motility
Z	0	0.0	Cytoskeleton
W	0	0.0	Extracellular structures
U	69	3.9	Intracellular trafficking and secretion
O	89	5.0	Posttranslational modification, protein turnover, chaperones
C	141	8.0	Energy production and conversion
G	62	3.5	Carbohydrate transport and metabolism
E	121	6.8	Amino acid transport and metabolism
F	49	2.8	Nucleotide transport and metabolism
H	107	6.0	Coenzyme transport and metabolism
I	36	2.0	Lipid transport and metabolism
P	103	5.8	Inorganic ion transport and metabolism
Q	12	0.7	Secondary metabolites biosynthesis, transport and catabolism
R	158	8.9	General function prediction only
S	119	6.7	Function unknown
-	630	28.4	Not in COGs
